# Efficacy and safety of ultrasonic circular cyclocoagulation with second-generation probe in glaucoma: A retrospective study

**DOI:** 10.1371/journal.pone.0227389

**Published:** 2020-01-24

**Authors:** Chloé Chamard, Vincent Daien, Hyosun Han, David S. Greenfield, Max Villain

**Affiliations:** 1 Department of Ophthalmology, Gui de Chauliac Hospital, Montpellier, France; 2 INSERM (Institut National de la Santé Et de la Recherche Médicale), Montpellier, France; 3 The Save Sight Institute, Sydney Medical School, The University of Sydney, Sydney, NSW, Australia; 4 Department of Ophthalmology, University of Miami Miller School of Medicine, Bascom Palmer Eye Institute, Palm Beach Gardens, Florida, United States of America; University Hospitals Cleveland, UNITED STATES

## Abstract

**Background:**

To assess the efficacy and safety of the second-generation probe of ultrasonic circular cyclocoagulation (UC3) in naive or refractory glaucoma, with a 6-month follow-up.

**Methods:**

A retrospective consecutive case-series study included patients having a UC3 procedure with the second-generation probe of the EyeOP1 device, intraocular pressure (IOP) ≥ 21 mmHg and under maximum tolerated medical treatment, with or without previous glaucoma surgery. Surgical success was defined at the 6-month post-operative visit as IOP > 5 and ≤ 21 mmHg with IOP reduction ≥ 20% from baseline, without any reoperation, and visual acuity better than negative light perception.

**Results:**

100 patients were included and 97 (97.0%; 97 eyes) attended the 6-month follow-up. At 6 months, surgical success was obtained in 48 eyes (49.5%). Intraocular pressure was reduced from a mean ± SD preoperative value of 28.0 ± 5.6 mmHg to 19.3 ± 7.1 mmHg at 6 months (p<0.0001). The proportion of eyes requiring oral acetazolamide decreased from 57.0% to 30.0% between baseline and 6 months after surgery (p = 0.0007). We observed 15 (15.0%) cases of postsurgical macular edema, 8 (8.0%) of hypotony, and 20 (20.0%) of visual acuity loss > 2 Snellen lines. Postsurgical macular edema was associated with a history of epiretinal membranes, uveitis or retinal detachment. Risk factors for hypotony were a history of diabetes or trabeculectomy.

**Conclusions:**

The second-generation UC3 probe significantly reduced IOP in eyes with naive and refractory glaucoma but severe post-operative complications were often observed. Further studies are needed to better identify responders and decrease the high risk for complications associated with the procedure.

## Introduction

Lowering intraocular pressure (IOP) is the only approach with proven effectiveness in preventing visual field deterioration and delaying the progression of glaucoma.[1–3] Although most surgical treatments aim at increasing aqueous humor evacuation, an alternative strategy is to reduce its production. Since the 1930s, many cyclodestructive techniques have been used, all with a high rate of potentially vision-threatening complications.[4–6] Hence, cyclodestructive procedures are usually reserved for refractory glaucoma in eyes with little or no visual potential.[7–9]

High-intensity focused ultrasound (HIFU) was first proposed as an alternative for lowering IOP in the 1980s.[6,10–12] Following recent breakthroughs in HIFU technology, a new cyclocoagulation device, the EyeOP1 (EyeTechCare, Rillieux-la-Pape, France), was developed for selective thermal coagulation of the ciliary body, sparing the adjacent ocular structures.

After experimental studies[13,14] and a clinical pilot,[15] several prospective clinical studies investigated ultrasonic circular cyclocoagulation (UC3) for refractory glaucoma.[16–19] In 2015, a multicenter study evaluated UC3 in 30 patients with primary open-angle glaucoma without previous filtering surgery.[20] All these studies supported the effectiveness of the procedure in reducing IOP while being safe.[15–20]

A second-generation probe was recently developed to boost clinical efficacy. It differs from the original mainly in its broader active transducer area (4 vs 2.5 mm), which allows for an average of 45% of the ciliary body circumference to be treated versus 30%. Only a few studies have evaluated this new device.[19,21] In 2017, a study from India reported similar success rates but high rates of anterior chamber inflammatory reaction and 3 cases of hypotony.[21]

The main objective of our study was to assess the efficacy of a second-generation UC3 probe in naive or refractory glaucoma. The secondary objectives were to assess the safety of the UC3 probe and to identify risk factors for severe vision-threatening complications such as macular edema and hypotony.

## Methods

This study followed the STROBE checklist for reporting observational study findings.[22]

### Design

Retrospective consecutive case-series study with 6-month follow-up. Examinations were performed before the procedure and 1 week, 1, 3, and 6 months afterwards. Preoperative hypotensive medications remained unchanged during the first month except for cases of hypotony, then adjusted if necesary during follow-up. Re-treatment was proposed after a minimum of 3 months if surgical success was not achieved. Patients were retreated with a second UC3 procedure, DL-CPC (diode laser cyclophotocoagulation) or trabeculectomy when IOP target was not reached, according to glaucoma stage, prior treatments and visual potential.

### Setting

The present study was performed in the department of ophthalmology in Montpellier University Hospital between September 2015 and May 2017. UC3 procedure is a routine care in the center since September 2015. All patients provided verbal and written informed consent for surgery and all data were fully anonymized before its access and analysis. The study was approved by the Montpellier University Hospital Institutional Review Board.

### Participants

We examined files from 100 consecutive patients aged ≥ 18 years with primary or secondary glaucoma, with IOP ≥ 21 mmHg and under maximum tolerated medical treatment, with or without previous filtering surgery. The eye was considered as refractory glaucoma if it required a UC3 procedure in spite of a history of trabeculectomy and/or sclerectomy and/or DL-CPC procedure and/or UC3 procedure.

### Data sources/measurements

#### Baseline examination

Baseline evaluation included medical history, slit-lamp biomicroscopy with gonioscopy and mydriatic fundus examination, Goldmann aplanation tonometry with 3 measurements, ultrasound pachymetry, best-corrected visual acuity (BCVA), automated visual field when applicable (Humphrey Field Analyzer; 24–2 SITA-standard program; Carl Zeiss Meditec, Dublin, CA) or Goldmann visual field when impossible, retinal optical coherence tomography (OCT) profile assessed by spectral-domain scanning laser ophthalmoscopy OCT (Spectralis Heidelberg Retinal Angiograph/OCT, Heidelberg Engineering GmbH), and ultrasound biomicroscopy (UBM) performed with a 50-MHz probe (Aviso; Quantel Medical, Clermont-Ferrand, France). Radial and transverse scans were obtained at 0°–180°, 45°–225°, 90°–270°, and 135°–315° meridians. We classified glaucoma severity based on the visual field mean deviation using the Hodapp-Parrish-Anderson grading scale: early glaucoma was defined as MD > -6dB, moderate glaucoma was defined as MD between -12dB and -6dB, and severe glaucoma was defined as MD < -12dB.[23] Patients followed with Goldman perimetry were classified as end-stage disease.

#### UC3 procedure

**Fig 1:**
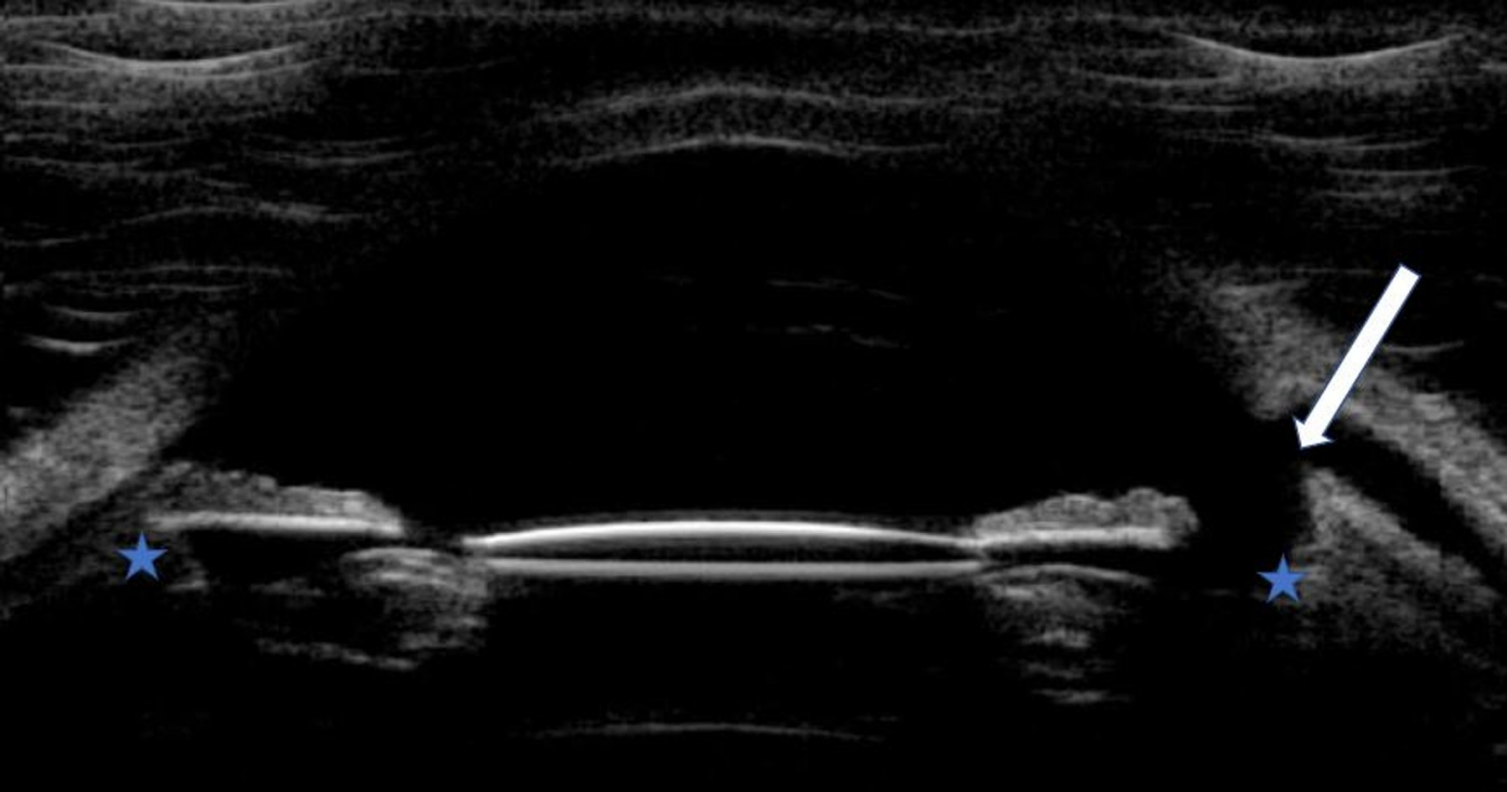
Ultrasound biomicroscopy vertical image in a refractory glaucoma (previous trabeculectomy). Blue stars, ciliary bodies; white arrow, trabeculectomy site.

UC3 procedures were mainly performed under general anesthesia or peribulbar when medical history of patients did not allow it. All UC3 procedures were performed with the EyeOP1 second-generation probe with an active transducer area of 4 mm. The choice between 3 different diameters of the device was based on UBM images. The location of the focal zones to be treated was simulated by using UBM images, and the model that best targeted the ciliary body was chosen (Fig 1).

The following parameters were used for all treatments: operating frequency, 21 MHz; number of sectors activated, 6; acoustic power, 2–3 W; duration of each of the 6 shots, 8 sec; time between shots, 20 sec; and total duration of the procedure, 2 min 32 sec. The coupling cone was placed so as to be centered and to avoid treating the nasal-temporal meridian. The HIFU device has been described in detail.[15,21]

#### Postoperative treatment

Participants received topical flurbiprofen 3 times daily for 4 weeks and rimexolone 3 times daily for 1 to 4 weeks.

#### Follow-up examinations

Follow-up included collection of symptoms, Goldmann aplanation tonometry, slitlamp biomicroscopy with fundus examination, and BCVA postoperatively at 1 week and 1, 3, and 6 months. Macular OCT was systematically performed at 1, 3, and 6 months.

### Main outcome measures

The primary outcome was surgical success defined at the 6-month post-operative visit as mean IOP > 5 and ≤ 21 mmHg with IOP reduction from baseline ≥ 20%, without any retreatment, and visual acuity better than negative light perception.[24] If a patient received additional treatment, such as filtering surgery or cyclodestruction (diode laser cyclophotocoagulation [DL-CPC]), to lower ocular pressure, the UC3 procedure was considered a failure and IOP values were not collected after the additional surgery. The primary outcome was analysed independently of the occurrence of any complication.

Secondary outcomes were vision-threatening complications as defined in the World Glaucoma Association “Guidelines on the design and reporting of glaucoma surgical trials”.[25,26]

### Statistical analysis

Descriptive statistics (mean ± SD, median [interquartile range]) were used to report demographic and ocular baseline characteristics. Quantitative variables were analyzed by Student *t* test or paired Student *t* test for paired data. Categorical variables were analyzed by chi-square test or Fisher test depending on the sample size. Data were adjusted on age and sex, but no multivariate analysis was possible. The risk factors for post-operative complications were assessed by univariate analysis. Odds ratios (ORs) and 95% confidence intervals (CIs) are reported. A survival analysis and Kaplan Meier representations were realised for severe post-operative complications. Statistical significance was set at p < 0.05. SAS Enterprise Guide 7.1 was used for data analysis.

## Results

### Patient characteristics

**Fig 2:**
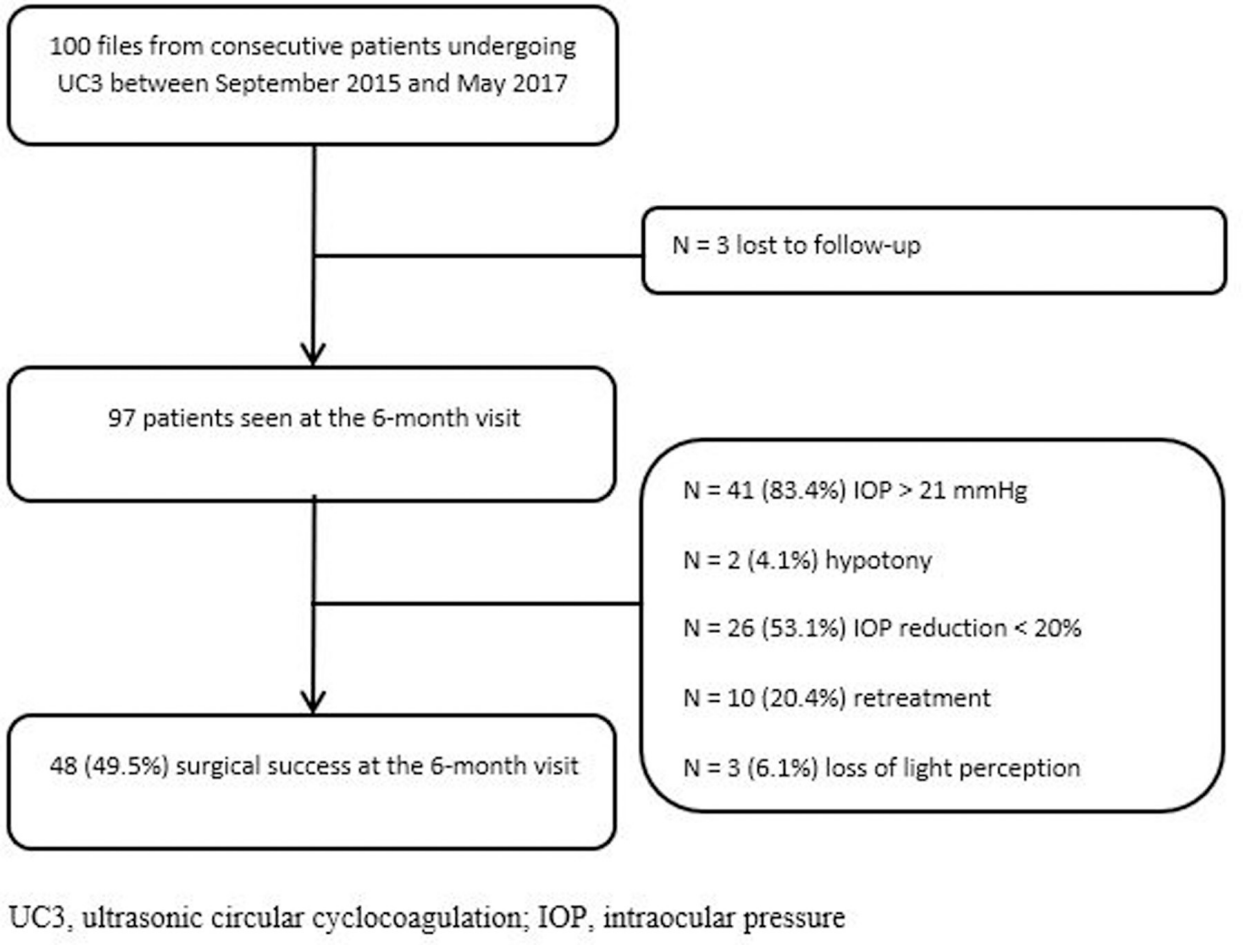
Study flowchart. UC3, ultrasonic circular cyclocoagulation; IOP, intraocular pressure.

A total of 100 patients (100 eyes) were included in the study, 55.0% were naive and the other 45.0% were refractory glaucoma. Mean (SD) number of prior surgical procedures in refractory glaucoma was 1.3 (0.9). Prior trabeculectomy, sclerectomy, UC3 procedure and DL-CPC were performed in 73.3%, 8.9%, 15.6% and 8.9% of patients, respectively. Six patients (6.3%) had early glaucoma, 20 (21.1%) moderate glaucoma, 35 (36.8%) severe glaucoma and 34 (35.8%) end-stage disease (follow-up with Goldman). Visual field missing data concerned the 5 remaining patients. Three patients were lost to follow-up, the other 97 patients were followed up to 6 months after surgery (M6). The flow of patients in the study is in Fig 2. Baseline characteristics of patients by surgical success and failure are summarized in Table 1.

**Table 1:** Baseline characteristics of patients according to surgical success^a^ or failure^b^.

	Success	Failure	OR (95% CI)	P value	Adjusted P value^c^
	N = 48 (49.5%)	N = 49 (50.5%)			
Age (years), median (IQR)	68 (64–80)	75 (61–81)	1.0 (1.0–1.0)	0.7	NA
Sex (men), n (%)	30 (62.5)	23 (46.9)	2.5 (1.1–5.5)	**0.05**	NA
Ethnicity, n (%)					
White	43 (89.6)	44 (89.8)	Reference		
Black	2 (4.2)	3 (6.1)	0.7 (0.1–4.2)	0.7	0.6
Other	3 (6.3)	2 (4.1)	1.5 (0.2–9.4)	0.7	0.9
Diagnosis, n (%)					
POAG	23 (47.9)	30 (61.2)	0.6 (0.3–1.4)	0.2	0.3
CACG	7 (14.6)	3 (6.1)	2.5 (0.6–10.5)	0.2	0.3
PXFG	5 (10.4)	6 (12.2)	0.8 (0.2–2.9)	0.8	0.8
PG	3 (6.3)	0 (0)	NA	0.2	1.0
Aphakic	4 (8.3)	0 (0)	NA	0.1	1.0
Uveitic	0 (0)	3 (6.1)	NA	0.2	1.0
Other causes (neovascular, traumatic, congenital, post-surgical)	6 (12.5)	7 (14.3)	0.8 (0.3–2.7)	0.8	0.7
Previous glaucoma surgery, n (%)					
Filtering surgery	19 (39.6)	14 (28.6)	1.6 (0.7–3.7)	0.4	0.3
Laser trabeculoplasty	13 (27.1)	17 (34.7)	0.7 (0.3–1.6)	0.5	0.4
DL-CPC	1 (2.1)	3 (6.1)	0.3 (0.03–3.2)	0.6	0.4
Ultrasound ciliary plasty	2 (4.2)	5 (10.2)	0.4 (0.07–2.0)	0.4	0.3
History, n (%)					
Diabetes	13 (27.1)	7 (14.3)	2.2 (0.8–6.0)	0.2	0.2
Uveitis	3 (6.3)	6 (12.2)	0.5 (0.1–2.0)	0.5	0.4
Vitrectomy surgery	7 (14.6)	7 (14.3)	1.0 (0.3–3.1)	1.0	0.8
Retinal detachment	4 (8.3)	5 (10.2)	0.8 (0.2–3.1)	1.0	0.7
Epiretinal membrane	11 (22.9)	11 (22.4)	1.0 (0.4–2.5)	1.0	0.9
Lens status, n (%)					
Phakic	17 (35.4)	19 (38.8)	Reference		
Pseudophakic	26 (54.2)	30 (61.2)	0.9 (0.4–2.1)	0.8	0.9
Aphakic	5 (10.4)	0 (0)	NA		
IOP, median (IQR)	26 (24–32)	26 (24–32)	1.0 (0.9–1.1)	0.4	0.7
Glaucoma eye drops, median (IQR)	4 (3–4)	3 (3–4)	1.6 (1.0–2.6)	**0.01**	0.06
Glaucoma systemic medications, n (%)	27 (54)	29 (60)	0.8 (0.4–1.8)	0.6	1.0
LogMAR BCVA, median (IQR)	0.3 (0.2–0.5)	0.3 (0.2–0.7)	0.5 (0.2–1.1)	0.4	0.1

NA, not available; POAG, primary open angle glaucoma; CACG, chronic angle closure glaucoma; PXFG, pseudo-exfoliative glaucoma; PG, pigmentary glaucoma; DL-CPC, diode laser cyclophotocoagulation; IOP, intraocular pressure; logMAR, log minimum angle of resolution; BCVA, best-corrected visual acuity; OR, odds ratio; 95% CI, 95% confidence interval; IQR, interquartile range.

^a^ IOP > 5 and ≤ 21 mmHg with IOP reduction ≥ 20% from baseline, without any reoperation, and visual acuity better than negative light perception.

^b^ IOP ≤ 5 or > 21 mmHg or IOP reduction < 20% from baseline, or a reoperation to lower IOP, or negative light perception visual acuity.

^c^ adjusted for age and sex.

### Efficacy

**Fig 3:**
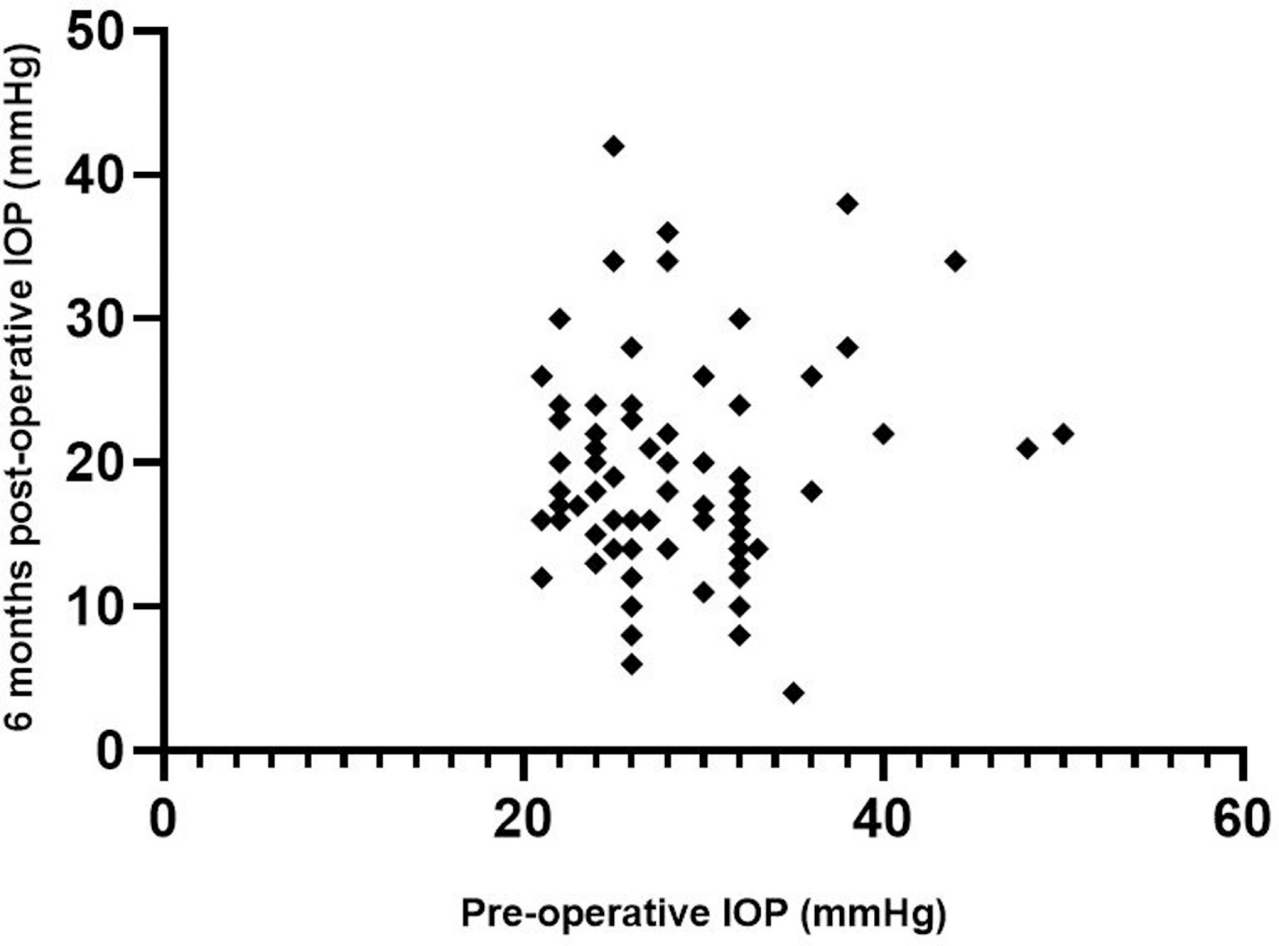
Scatter plot showing 6-month post-operative IOP according to pre-operative IOP. IOP, intraocular pressure.

Surgical success was obtained in 48/97 (49.5%) eyes at 6 months after surgery, among which 26 (54.2%) naive and 22 (45.8%) refractory glaucoma (p = 0.9). The proportion of efficacy was similar in different severity groups. Surgical success was obtained in 25.0%, 50.0%, 52.9% and 50.0% in early, moderate, severe and end-stage glaucoma, respectively (p = 0.9). The reasons for failure were post-operative IOP > 21 mmHg for 41 (83.4%) patients, hypotony for 2 (4.1%) patients, IOP reduction < 20% for 26 (53.1%) patients, retreatment for 10 (20.4%) patients and loss of light perception for 3 (6.1%) patients. For 31/97 (32.0%) patients, IOP-reduction was < 10% at M6. Sex and preoperative glaucoma eye drops were factors associated with surgical success on univariate analysis. When data were adjusted on age and sex, we found no statistically significant difference between success and failure groups. A second UC3 procedure was performed within 6 months in 6/97 (6.2%) patients: 2 who did not respond to the first treatment (33%) responded to the second treatment (IOP reduction > 20%). At 6 months, 10/97 (10.3%) patients required a second glaucoma surgery for insufficient response to HIFU treatment. These treatments occurred at least 2 months after the UC3. The mean IOP decreased from 28.0 ± 5.6 to 19.3 ± 7.1 mmHg between baseline and M6. In the group with surgical success, the IOP decreased from 28.0 ± 4.8 to 15.2 ± 3.4 mmHg from baseline to M6, with a relative IOP reduction of 45.7% (Table 2 and Fig 3). The mean number of topical hypotensive medication classes was significantly lower at M6 than baseline (3.1 ± 1.0 vs 3.2 ± 0.9; Student paired test p = 0.06). The percentage of eyes requiring oral acetazolamide decreased significantly from 57/100 (57.0%) to 27/87 (31.0%) between baseline and M6 (p = 0.0007) (Table 2).

**Table 2:** Intraocular pressure and hypotensive medications at baseline and follow-up.

	All patients				Success				Failure			
	N	IOP, mean (SD) [mean no. of glaucoma eyedrops]	Relative IOP reduction (%)	Systemic hypotensive medication, n (%)	N	IOP, mean (SD) [mean no. of glaucoma eyedrops]	Relative IOP reduction (%)	Systemic hypotensive medication, n (%)	N	IOP, mean (SD) [mean no. of glaucoma eyedrops]	Relative IOP reduction (%)	Systemic hypotensive medication, n (%)
**Baseline**	100	28.0 (5.6) [3.2]	-	56 (57)	50	28.0 (4.8) [3.4]	-	27 (54)	50	28.0 (6.4) [3.0]	-	29 (59)
**Day 1**	51	23.1 (7.2) [NA]	17.5	NA	20	20.9 (7.4) [NA]	25.4	NA	31	24.6 (6.8) [NA]	12.1	NA
**Day 8**	94	15.6 (5.7) [NA]	44.2	NA	49	14.2 (4.0) [NA]	49.3	NA	45	17.0 (6.8) [NA]	39.3	NA
**Month 1**	94	18.1 (9.4) [2.8]	35.4	NA	46	15.4 (7.3) [2.8]	45.0	NA	48	20.6 (10.5) [2.8]	26.4	NA
**Month 3**	91	18.1 (8.2) [3.1]	35.4	32 (34)	46	14.6 (5.3) [3.1]	47.9	16 (34)	45	21.7 (9.1) [3.0]	22.5	16 (35)
**Month 6**	87	19.3 (7.1) [3.1]	31.0	27 (30)	48	15.2 (3.4) [3.1]	45.7	14 (30)	39	24.4 (7.1) [3.0]	12.9	16 (31)

IOP, intraocular pressure; NA, not available. NB: the number of patients with IOP data collected at Month 6 was 87: 3 lost to follow-up and 10 retreated (IOP not collected after additional surgery)

### Safety

#### Mild and moderate postoperative complications

Complications included anterior chamber reactions, observed in 64 (64.0%) patients. In most cases, the reaction was mild and was resolved at 1 month. Minor pupil irregularities were frequent (39 [39.0%] patients), appearing just after the procedure, with an improvement over months. Induced astigmatism occurred in 11/88 (12.5%) patients and improved over months. Cataract progression was noted in 7/37 (18.9%) patients.

#### Serious postoperative complications and their risk factors

**Fig 4:**
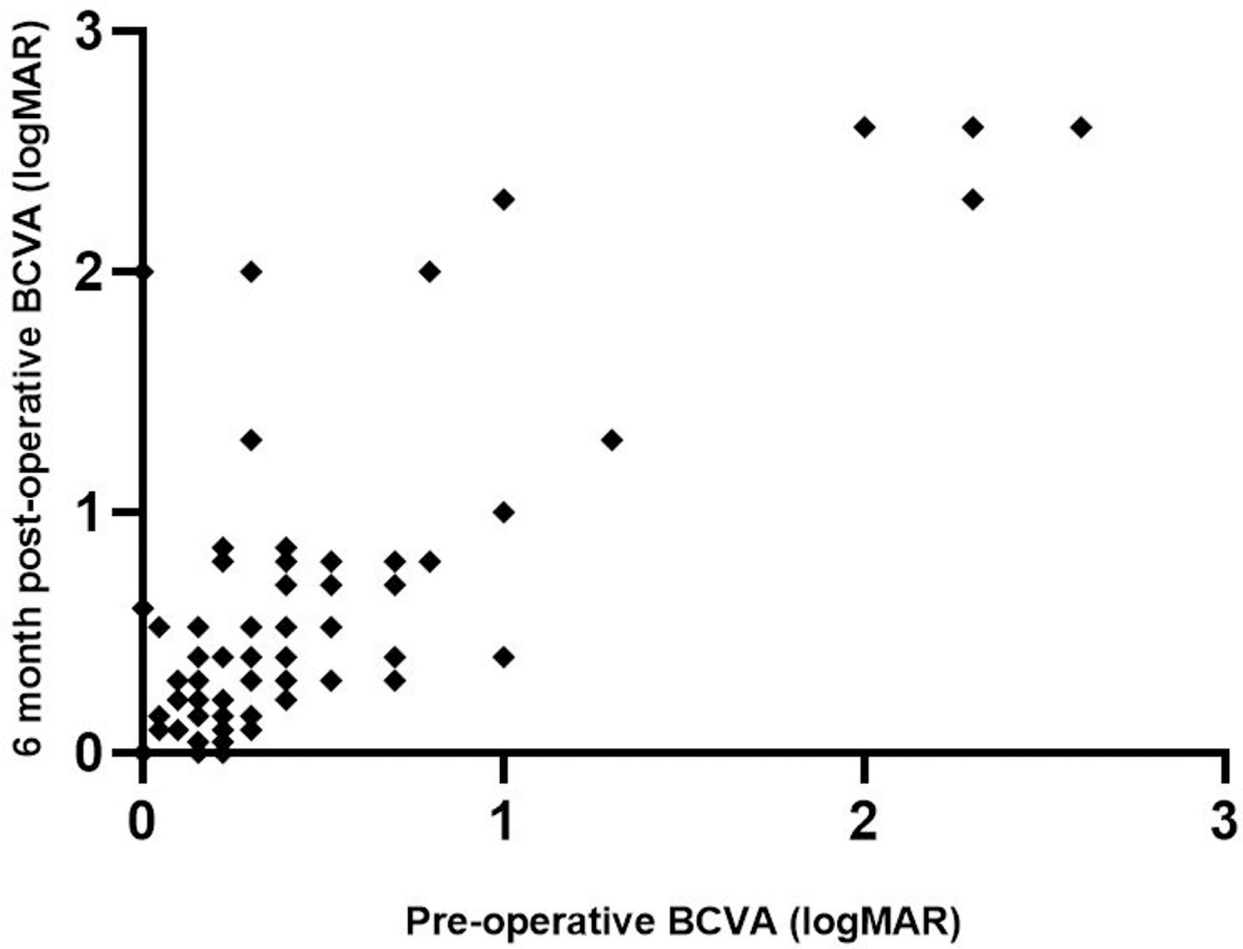
Kaplan-Meier plots representing the probability of being free of hypotony (A), macular edema (B) and > 2 Snellen lines of visual loss (C) over time.

During follow-up, 8 (8.0%) patients required hospitalization because of IOP spikes (n = 3), hypotony with choroidal detachment (n = 2), retinal and choroidal detachment (n = 1), infectious keratitis (n = 1), and ischemic retinal vein occlusion (n = 1).Macular edema, diagnosed on OCT, developed in 23/100 (23.0%) patients: 4 cases occurred in association with hypotony; 1 patient had ischemic central retinal vein occlusion occurring 3 months after the procedure; 1 patient showed recurrence of cystic macular edema in the context of known retinal arteriovenous malformations; 1 patient had exudative edema due to myopic choroidal neovascularization at 3 months and 1 patient had focal diabetic macular edema. In the 15 remaining patients, macular edema was not associated with any other retinal disorder and occurred without previous macular diseases except for vitreomacular interface disorder and epiretinal membrane. We assume that these 15 cases were related to postsurgical edema from the UC3 procedure, among which 8 (53.3%) naive and 7 (46.7%) refractory glaucoma (p = 1.0). Six of 15 (40.0%) patients showed persistent macular edema at M6. In these 15 patients, mean visual acuity at baseline was 0.5 ± 0.6 logMAR, decreasing to 0.6 ± 0.5 logMAR at M1 and 0.7 ± 0.7 logMAR at M6. The Kaplan-Meier plots representing survival without significant visual loss are presented in Fig 4.

Univariate analysis revealed increased probability of postsurgical macular edema with pre-existant epiretinal membrane (OR = 5.6, 95% CI [1.7–17.8], p = 0.004), history of uveitis (9.9 [2.3–43.0], p = 0.002) and history of retinal detachment (5.7 [1.3–24.7], p = 0.02) (Table 3).

**Table 3:** Risk factors for developing postsurgical macular edema and hypotony.

	Postsurgical macular edema (n = 15)	No postsurgical macular edema (n = 85)	OR (95% CI)	P value
Age, median (IQR)	67 (54–80)	73 (64–81)	1.0 (1.0–1.0)	0.3
Sex (men), n (%)	9 (60)	43 (50)	1.4 (0.5–4.4)	0.5
Diabetes, n (%)	0 (0)	19 (23)	NA	1.0
Ophthalmologic history, n (%)				
Retinal detachment	4 (27)	5 (6)	5.7 (1.3–24.7)	**0.02**
Uveitis	5 (33)	4 (5)	9.9 (2.3–43.0)	**0.002**
Vitrectomy surgery	4 (27)	10 (12)	2.7 (0.7–10.1)	0.1
Epiretinal membrane	8 (53)	14 (17)	5.6 (1.7–17.8)	**0.004**
Trabeculectomy	4 (27)	29 (35)	0.7 (0.2–2.4)	0.6
Deep sclerectomy	2 (13)	2 (2)	6.3 (0.8–48.8)	0.08
Central retinal vein occlusion	0 (0)	1 (1)	NA	1.0
Preoperative medication, n (%)				
Prostaglandin analogs	13 (93)	79 (94)	0.8 (0.09–7.6)	0.9
Oral acetazolamide	9 (64)	46 (55)	1.5 (0.5–4.8)	0.5

NA, not available.

We observed 8 (8.0%) cases of hypotony (IOP ≤ 5 mmHg), among which 1 (12.5%) naive and 7 (87.5%) refractory glaucoma (p = 0.02). Six of these showed choroidal detachment. One case of choroidal detachment was associated with retinal detachment, occurring within 2 months of the procedure. Successful vitreoretinal surgery was performed, during which partial ciliary body detachment was observed. Hypotony was transient in all cases but one and was resolved within 2 months. In the remaining case, choroidal detachment spontaneously resolved. No case of phthisis bulbi was observed during the 6-month follow-up. Kaplan-Meier plots are in Fig 4.

Univariate analysis revealed increased probability of hypotony with a history of diabetes (OR = 4.8, 95% CI [1.1–21.0], p = 0.05) or trabeculectomy (7.2 [1.4–38.1], p = 0.01) (Table 4).

**Table 4:** Risk factors for developing postsurgical hypotony.

	Postsurgical hypotony (n = 8)	No postsurgical hypotony (n = 92)	OR (95% CI)	P value
Age, median (IQR)	73 (61–83)	73 (63–80)	1.0 (1.0–1.1)	0.7
Sex (men), n (%)	5 (63)	48 (52)	1.5 (0.3–6.8)	0.7
Diabetes, n (%)	4 (50)	16 (17)	4.8 (1.1–21.0)	**0.05**
Ophthalmologic history, n (%)				
Retinal detachment	0 (0)	9 (10)	NA	1.0
Vitrectomy surgery	0 (0)	14 (15)	NA	0.6
Trabeculectomy	6 (75)	27 (29)	7.2 (1.4–38.1)	**0.01**
Deep sclerectomy	1 (13)	3 (3)	4.2 (0.4–46.3)	0.3
Transscleral diode laser cyclocoagulation	0 (0)	4 (4)	NA	1.0
Angle-closure	3 (38)	7 (11)	4.6 (0.9–23.7)	0.08
Preoperative medication, n (%)				
Oral acetazolamide	5 (63)	51 (56)	1.3 (0.3–5.8)	1.0

NA, not available.

**Fig 5:**
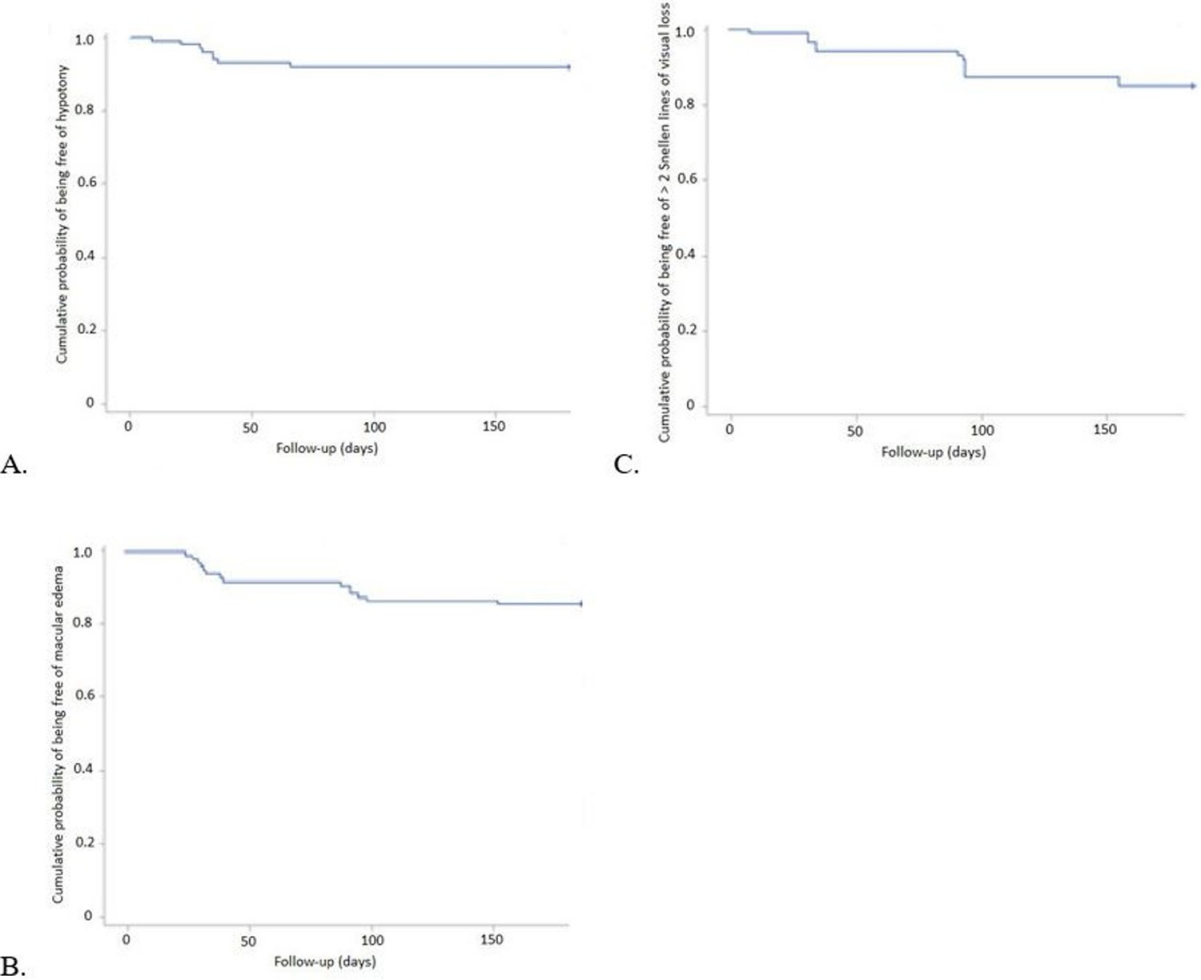
Scatter plot showing 6-month post-operative BCVA according to pre-operative BCVA. BCVA, best corrected visual acuity; logMAR, log minimum angle of resolution.

A scatter plot representing 6-month post-operative BCVA according to pre-operative BCVA is in Fig 5. Mean visual acuity was significantly reduced from baseline logMAR BCVA, from 0.50 ± 0.61 to 0.63 ± 0.70 at M6 and 0.64 ± 0.70 at last follow-up (p<0.001). A total of 20 (20.0%) patients had visual acuity loss > 2 Snellen lines at M6, among which 6 (30.0%) naive and 14 (70.0%) refractory glaucoma (p = 0.03); 13 had visual acuity loss > 4 Snellen lines, which was attributed to cataract progression (n = 1), corneal decompensation (n = 2), choroidal detachment/hypotony maculopathy (n = 2), ischemic central retinal vein occlusion (n = 1), postsurgical edema (n = 2), agonic glaucoma progression (n = 3), underlying macular disease (n = 1), and infectious corneal ulcer (n = 1). The Kaplan-Meier plots are in Fig 4.

#### Success rate without severe complications

In 32% of patients, surgical success was obtained without severe complications (macular edema, hypotony and loss of visual acuity > 2 Snellen lines).

## Discussion

This study was conducted to evaluate the efficacy and safety of second-generation UC3 in primary or secondary glaucoma naive or refractory to filtering surgery. The surgical success rate was 49.5% (48/97 eyes) at 6 months after surgery, with a decrease in topical and systemic glaucoma medication. Serious post-operative complications were macular edema in 15.0% of eyes, hypotony in 8.0% and visual acuity loss > 2 Snellen lines in 20.0%.

Previous studies reported encouraging efficacy results, with success rates ranging from 57.0% to 83.3% and IOP percentage decreases ranging from 26.0% to 38.0%. The IOP percentage decrease in the present study is comparable to that in previous studies: 31.0% mean reduction at 6 months. Overall, 60.8% of patients showed an IOP decrease ≥ 20%, with a mean IOP decrease of 43.5% in these patients. Although significant, these levels of reduction may not be sufficient to achieve a target IOP ≤ 15 mmHg in patients with preoperative IOP > 27 mmHg. Approximately one third of our patients did not respond to treatment, with IOP reduction < 10%. The ultrasound beam may not have reached the target organ if the probe diameter was incorrectly determined by preoperative UBM imaging. This theory is supported by our observation that when re-treatment was required, UBM imaging was repeated, which resulted in a change of probe size in 2 of 6 cases. In those 2 patients, the second procedure was fully successful. Inadequate centering or intraoperative displacement of the coupling cone (which is positioned and handled by the operator) may also result in poor efficacy. Micropulse CPC (MP-CPC) seems more efficient than UC3 in refractory glaucoma, inducing more than 40% reduction in IOP at 12 months after surgery but reduced efficacy with time.[27] DL-CPC allows for a 44% mean reduction in IOP at 19 months in eyes with higher baseline IOP (36 mmHg).[28]

In all previous major studies, the procedure was reported as safe without serious intra- or postoperative complications.

Deb-Joardar and Reddy[21] reported postoperative anterior chamber reactions in 92% of patients with the second-generation probe. In the present study, reactions were noted in 64.0% of patients. The second-generation probe may cause more inflammatory reactions than the first-generation probe by increasing the surface area and duration of exposure to the ultrasound beam.[8,21] UC3 procedure might not be indicated in uveitic glaucoma because of inefficiency. In the present study, all uveitic glaucoma were considered as failure: 1 for significant loss of vision due to post-operative macular edema and the 2 others needed a retreatment to reach the target IOP.

We observed high rates of pupil modification (39.0%) and induced astigmatism (12.5%). This clinical observation was poorly documented and probably underestimated in previous studies, with 15% pupil modification and 2.9% astigmatism rates in Aptel et al and Deb-Joardar and Reddy, respectively.[20,21] Thermal elevation may cause ciliary muscle and iris root damage. Furthermore, focal shrinkage of scleral tissue could result in sectoral pupillary distortion and induced astigmatism. Further analysis could be worthwhile, including pre- and postoperative keratometry and corneal topography.

Postsurgical macular edema rate was higher in the present study than in previous studies (15.5% vs 1.4–3.3%).[18,20,21] First, macular edema occurrence may have been underestimated in previous studies because of insufficient screening with macular OCT. We systematically performed macular OCT and observed clinically relevant macular edema with decreased visual acuity in 60% of cases. We assume that this complication could easily be missed. The prevalence of history of uveitis (9.0%), epiretinal membrane (22.7%), vitreoretinal surgery (14.0%), and choroidal detachment (9.0%) was higher in our cohort than in the general population, and these characteristics were not systematically recorded in previous studies. Prophylactic postoperative treatment with topical non-steroidal anti-inflammatory drugs seems justified. We also recommend screening for underlying macular diseases and ocular inflammatory history before UC3 treatment. The indications for UC3 need to be weighed against any history of uveitis, epiretinal membrane or retinal detachment.

We observed 6 cases of hypotony with choroidal detachment, including one case associated with retinal detachment, which has not been reported previously with UC3, and 2 cases of hypotony without choroidal detachment. The combined rate for these severe complications was 8%. In previous cohorts, the combined rate for these events was 2.5% (5 cases/203 patients). Deb-Joardar and Reddy reported a rate of 4.1% hypotony with the second-generation probe.[21] This variability may be explained by small sample sizes, but it also suggests that the complication could be promoted by use of the second-generation probe. In our series, B-scan ultrasonography findings confirming the serous nature of choroidal detachments and a case of exudative retinal detachment strongly suggest uveal effusion. In our analysis, the risk factors for hypotony were a history of diabetes or trabeculectomy. Diabetes could promote hypotony and choroidal detachment by increasing choriocapillaris permeability and trabeculectomy with multiple hypotensive surgeries that modify scleral architecture. Further studies analyzing axial length and scleral thickness might be of interest to understand the mechanism of such hypotony.

In previous cohorts with the first-generation probe treatment, mean baseline visual acuity was usually lower (0.84–1.94 logMAR), with more recent studies increasingly including patients with higher visual potential. Visual acuity loss > 2 Snellen lines was observed in 20.0% of cases. Deb-Joardar and Reddy[21] reported visual acuity loss ≥ 2 lines in 8.2% of patients with the second-generation probe. Our high rate of visual acuity deterioration was related to our higher rate of vision-threatening complications.

When comparing the safety of UC3 to DL-CPC procedures, MP-CPC induces less hypotony, with a 6% rate, but leads to more visual acuity loss, 26%, at 3 months. Assessing the effect of DL-CPC on visual acuity is difficult because this treatment is often used for lost eyes, but it results in 4% of phtisis bulbi, which is intolerable in functional eyes.[28]

The small sample size and short follow-up are the main limitations of our study. Although it was a retrospective study, the follow-up was standardized with systematic recording of data and complications. Also, the surgical procedure was standardized and allowed for fair comparison with other data concerning the second-generation UC3 probe.

## Conclusion

In previous studies mostly evaluating the first-generation probe, UC3 of the ciliary body seemed an encouraging emerging technique, with efficacy for reducing IOP and a good safety profile extending its indication to patients naive of filtering surgery. Our study, conducted with the second-generation probe, corroborates previous efficacy findings. However, we report a higher rate of severe complications that might reduce or threaten visual function. These complications should be considered carefully before choosing this treatment, especially in patients naive of filtering surgery and with good visual potential. Because the second-generation probe seems to cause more inflammatory complications, including macular edema, a history of uveitis and epiretinal membrane might be carefully searched and UC3 avoided in this situation. The risk/benefit balance must be assessed before enrolling patients with prior trabeculectomy for a UC3 procedure because of a highly increased risk of hypotony. Further long-term studies with larger populations are needed to better identify responders, risk factors for complications, and an effective impact on visual function.

## Supporting information

S1 Table Database. (CSV)
